# Role of a 193 nm ArF Excimer Laser in Laser-Assisted Plasma-Enhanced Chemical Vapor Deposition of SiN*_x_* for Low Temperature Thin Film Encapsulation

**DOI:** 10.3390/mi11010088

**Published:** 2020-01-13

**Authors:** Kunsik An, Ho-Nyun Lee, Kwan Hyun Cho, Seung-Woo Lee, David J. Hwang, Kyung-Tae Kang

**Affiliations:** 1 Micro/Nano Process Group, Korea Institute of Industrial Technology (KITECH), Ansan 15588, Korea; kunsik1214@kitech.re.kr (K.A.); khcho@kitech.re.kr (K.H.C.); 2 Surface Technology Group, Korea Institute of Industrial Technology (KITECH), Incheon 21999, Korea; hnlee@kitech.re.kr; 3 Department of Mechanical Engineering, Hanyang University, Seoul 04763, Korea; randy7406@naver.com; 4 Department of Mechanical Engineering, State University of New York, Stony Brook, NY 11794, USA

**Keywords:** laser assisted plasma enhanced chemical vapor deposition, silicon nitride, low temperature encapsulation, silane photolysis, ArF excimer laser

## Abstract

In this study, silicon nitride thin films are deposited on organic polyethylene-naphthalate (PEN) substrates by laser assisted plasma enhanced chemical vapor deposition (LAPECVD) at a low temperature (150 °C) for the purpose of evaluating the encapsulation performance. A plasma generator is placed above the sample stage as conventional plasma enhanced chemical vapor deposition (PECVD) configuration, and the excimer laser beam of 193 nm wavelength illuminated in parallel to the sample surface is coupled to the reaction zone between the sample and plasma source. Major roles of the laser illumination in LAPECVD process are to compete with or complement the plasma decomposition of reactant gases. While a laser mainly decomposes ammonia molecules in the plasma, it also contributes to the photolysis of silane in the plasma state, possibly through the resulting hydrogen radicals and the excitation of intermediate disilane products. It will also be shown that the LAPECVD with coupled laser illumination of 193 nm wavelength improves the deposition rate of silicon nitride thin film, and the encapsulation performance evaluated via the measurement of water vapor transmission rate (WVTR).

## 1. Introduction

Low temperature deposition of dielectric thin films has been a topic of extensive research in various application fields that rely on heat and/or damage vulnerable materials. Among materials for the dielectric thin films, silicon nitride (SiN_x_) has secured significant attention in various application fields including insulating layers in capacitor [1,2,3], gate insulators for thin film transistor [4,5,6], and encapsulation layers on electronic devices [7,8]. While a variety of deposition methods have been employed to deposit silicon nitride, including sputtering [9,10], atomic layer deposition (ALD) [11,12,13], and chemical vapor deposition (CVD) [14,15] the conventional methods still have technical limitations for low temperature encapsulation, whereby sputtering leaves a host of defects, the deposition rate of ALD is too low to be commercialized, and traditional CVD requires high process temperature condition to decompose reactant gases. To overcome these limitations of conventional methods, diverse variants of CVD for low temperature processes have been developed such as metalorganic chemical vapor deposition (MOCVD) [16,17], oxidative chemical vapor deposition (oCVD) [18], and so on. MOCVD utilizes a metalorganic vapor as a precursor for semiconductor compounds and oCVD is a deposition method for conjugated conducting polymers using condensation polymerization of monomer gases at temperatures even lower than 100 °C.

Plasma-enhanced chemical vapor deposition (PECVD) [19,20,21,22] and laser-assisted chemical vapor deposition (LACVD) [23,24] are also CVD variants that require lower temperatures than traditional CVD by adding a plasma or laser source. Laser-assisted plasma-enhanced chemical vapor deposition (LAPECVD), attempted more recently, is a unique combination of such developments that utilizes both laser and plasma to assist the relevant chemical reactions. In LAPECVD, the role of the laser is mainly differentiated by the wavelength or photon energy of laser beam as in LACVD. Tsai et al., [25,26] and Choi et al. [27] studied the use of LAPECVD to deposit of SiN_x_ thin films with an infrared CO_2_ laser beam. They found that the infrared laser beam cannot directly participate in the chemical reaction at a practical beam size needed to cover a large sample area. Although surface heating by a laser beam illuminated onto the sample enabled the formation of denser film with reduced hydrogen concentration, it is not consistent with the main objective of the current study due to the undesirable sample heating effect. On the other hand, excimer laser beams of ultraviolet wavelength have been efficiently used to directly decompose the reactant gases in the LACVD configuration due to the high photon energy exceeding the bond energy of reactant gases [28]. In depositing SiN_x_ thin films with silane and ammonia, silane is mostly transparent to ArF excimer laser beams of 193 nm wavelength, however ammonia is strongly absorbing, and thus amidogen and hydrogen radicals are produced at the early stage, subsequently decomposing silane into silyl radicals and ultimately depositing SiN_x_ thin films. However, LAPECVD with ultraviolet wavelengths has not been reported to date.

In this study, we report the role of an ArF excimer laser beam of 193 nm wavelength in depositing SiN_x_ thin films by LAPECVD based on silane-ammonia gas mixtures towards encapsulation of polyethylene-naphthalate (PEN) flexible substrates. The ArF excimer laser was selected in this study because the photon energy of the laser should be larger than the decomposition energy of the reactant gases. Among the reactant gases, though photon energy of the 193 nm wavelength is not sufficient to decompose silane, we also revealed that the laser participated in the decomposition reaction of silane and increased the deposition rate. Participation of the ArF excimer laser in the decomposition of reactant gases is investigated by measuring *in-situ* the laser extinction intensity. The laser extinction intensity is also measured for each reactant gas with variable reactant gas pressure and plasma power levels so that role of laser can be clarified. The deposition rate and refractive index of deposited SiN_x_ thin films by LAPECVD and PECVD are compared to confirm the contribution of coupled laser beam to the reaction process. Finally, the encapsulation performance is evaluated through water vapor transmission rate (WVTR) measurements with SiN_x_ thin films deposited on PEN substrates.

## 2. Materials and Methods 

**Figure 1:**
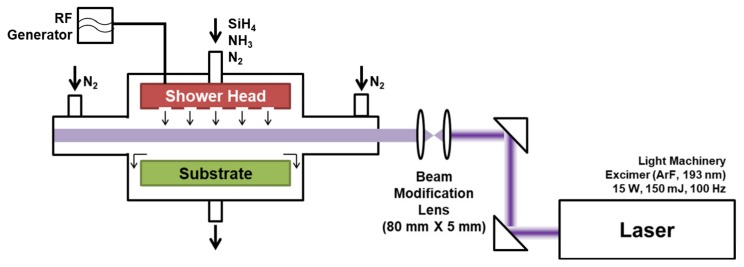
Schematic illustration of experimental setup for laser assisted plasma enhanced chemical vapor deposition (LAPECVD) of SiN_x_ thin films.

Figure 1 shows a schematic illustration of the experimental apparatus for LAPECVD. A PECVD process chamber was customized to accommodate the laser illumination. The process chamber includes a plasma generator and a sample stage, and the plasma power is adjustable up to 1000 W. Silane (SiH_4_, 100%) and ammonia (NH_3_, 100%) as reactant gases, and nitrogen (N_2_) as a buffer gas, were introduced into the chamber through separated lines, and were uniformly injected over the substrate via a shower head. Flow rates were separately controlled by mass flow controllers. Nitrogen (N_2_) buffer gas was separately delivered to the neighborhood of laser windows to prevent the window deposition. 

ArF excimer laser of 193 nm wavelength, 150 mJ pulse energy and 100 Hz repetition rate (IPEX-740, Light Machinery, Nepean, ON, Canada) was used for the LAPECVD. Before coupling to the process chamber, the laser beam was shaped into a rectangular beam of 80 mm in width (parallel to sample surface) and 5 mm in height (normal to sample surface) using a cylindrical beam expander set. The collimated laser beam was delivered into the chamber in parallel to the sample surface with the adjusted gap of 5 mm from the sample surface so that the organic substrate is free from direct photodegradation effects a while reasonable contribution of laser to the deposition process is made. The distance from the window glasses to the sample stage was 30 cm. For all the LAPECVD experiments in this study, pulsed energy, repetition rate and beam path were fixed. Unthreaded laser beam from the laser window was guided to the secondary window located at the opposite side of process chamber to avoid deposition at random sites within the chamber. For the measurement of laser extinction intensity after passing through the reaction zone, an aperture was installed at a location in the incoming laser path, i.e., before the laser beam reaches the chamber, to limit the beam size being smaller than the detector area. Then, the laser extinction intensity was measured at a location where laser beam escaped through the secondary window, and the normalized laser extinction intensity (*I_Ext_*) was estimated by dividing by the laser intensity measured in a vacuum state.

Eight-inch silicon wafers were used as sample substrates to characterize deposited silicon nitride thin films at a fixed substrate temperature of 150 °C. The distance between the showerhead and the sample stage was adjusted to 22 mm. Teonex Q65 was used for the PEN flexible polymer substrate which was purchased from Teijin Dupont Films (Kota Tangerang, Indonesia). The thickness of deposited silicon nitride thin film was measured by cross-sectional scanning electron microscopy (SEM, S-4800, Hitachi, Tokyo, Japan). Refractive index of the deposited thin films was measured by Filmetrics F20 (KLA Corporation, CA, USA), also confirming the film thickness. Flexible PEN substrates were also used to analyze the encapsulation performance of deposited thin film which was evaluated by the water vapor transmission rate (WVTR) analysis (Permatran W-700, Mocon, Brooklyn Park, MN, USA).

## 3. Results

**Figure 2:**
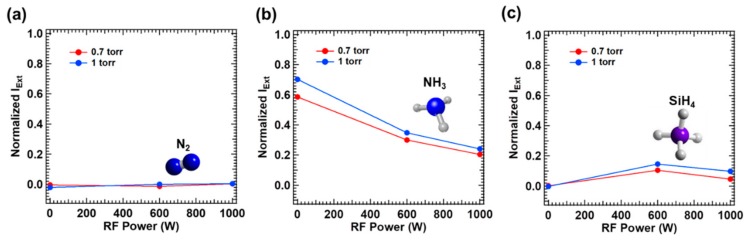
Normalized extinct laser power by propagation in the CVD chamber according to simultaneously generated radio frequency (RF) power with different gas molecules of (**a**) N_2_, (**b**) NH_3_ and (**c**) SiH_4_.

In order to investigate the mechanism of photodecomposition by the excimer laser of 193 nm wavelength, the normalized laser extinction intensity vs. vacuum state, was measured after filling the process chamber with N_2_, NH_3_, SiH_4_, respectively for varying plasma power levels as shown in Figure 2. To measure the laser extinction by pure N_2_ gas, N_2_ gas was supplied through the gas shower at the flow rate of 750 sccm with a separate N_2_ gas supplied to the laser window at 1000 sccm, and total pressure was adjusted to 0.7 and 1.0 torr via the exhaust valve control. As seen from Figure 2a, the extinction of excimer laser beam of 193 nm wavelength, i.e., combined absorption and scattering, was negligible (<0.01) upon filling N_2_ gas at the total pressure of 1 torr or lower, irrespective of plasma power. (Experiment 1 and 2 in Table 1)

However, the significant extinction of the laser occurred by ammonia gas filled at the total pressure of 0.7 and 1 torr as shown in Figure 2b (Experiment 3 and 4: NH_3_ gas was supplied through the gas shower at the flow rate of 150 sccm with a separate N_2_ gas supplied to the laser window at 1000 sccm). It is well known that ammonia can be efficiently decomposed by 193 nm wavelength; a single photon energy of ~6.4 eV exceeds the bond energy relevant to the following decomposition process (~5.6 eV) [29,30]:

(1) $$NH_{3}+h\nu \to NH_{2}+H$$

Although the use of a higher laser intensity, i.e., a tighter laser focuses in the current experimental configuration, led to decomposition to NH level as confirmed by optical emission spectrum measurements, the intensity of enlarged beam of 80 mm × 5 mm dimension decomposed to NH_2_ level [20].

It is important to note that the laser extinction decreased with plasma activation; e.g., the normalized laser extinction drops to 0.241 with plasma of 1000 W power vs. 0.703 without plasma, upon filling ammonia at the total pressure of 1 torr. It is believed that such decrement in the laser extinction with plasma is related to an effective reduction of the partial pressure of ammonia, deducting a decomposed portion by plasma. Anticipated ammonia decomposition processes by plasma are listed in Equation (2) [29] together with subsequent reactions in Equation (3) [28] via participation of H radicals generated. The byproducts of plasma induced decomposition and reaction processes shown in Equations (2) and (3) appear to be transparent to 193 nm wavelength, i.e., the bond energy levels of the byproducts exceed the 193 nm laser photon energy.

(2a) $$NH_{3}+plasma\to NH_{2}+H$$

(2b) $$NH_{2}+plasma\to NH+H$$

(2c) NH+plasma→N+H

(3a) $$H+NH_{3}\to H_{2}+NH_{2}$$

(3b) $$H+NH_{2}\to H_{2}+NH$$

(3c) $$H+NH\to H_{2}+N$$

When the total pressure was reduced to 0.7 torr, the laser extinction was reduced to 0.204 by the plasma power of 1000 W vs. 0.241 at the total pressure of 1 torr. Assuming that the contribution of scattering is negligible, the laser absorption by a participating gas medium follows Equation (4) [31], where *N* and *n* denote the number density of photons and molecules, respectively, *σ* is the absorption cross section determined by the relevant photon energy and molecular species, and *x* is the distance laser beam propagates through the medium. Thus, the absorbed number of photons during the laser beam propagation through a medium filled with participating molecules (i.e., ammonia) will increase as the number density of molecules, or the partial pressure of ammonia, increases:

(4) $$\frac{dN}{dx}=-Nn\sigma$$

Figure 2c shows the normalized laser extinction in silane (Experiments 5 and 6: SiH_4_ gas was supplied through the gas shower at the flow rate of 750 sccm with a separate N_2_ gas supplied to the laser window at 1000 sccm). Without plasma, no laser extinction was detected, which is an anticipated trend due to the bond energy of silane molecule >~8.0 eV, well exceeding the photon energy of 193 nm wavelength. Although the coherent two-photon absorption is possible at sufficiently high laser intensity [32], a mild laser intensity level used in this study could not induce two-photon absorption. With plasma activated, however, noticeable laser extinction was detected. In the plasma state, silane molecules are decomposed into SiH_3_ and H radicals whose reactivity is extremely high. Those radicals subsequently participate in the series of reactions that produce multiple species of hydrogenated silicon molecules and importantly disilane (Si_2_H_6_) [28,33]. The reactions to produce SiH_3_ and secondary reactions to generate Si_2_H_6_ species assisted by plasma are described by Equations (5) [29] and (6) [28,33], respectively.

(5a) $$SiH_{4}+plasma\to SiH_{3}+H$$

(5b) $$H+SiH_{4}\to H_{2}+SiH_{3}$$

(6a) $$2SiH_{3}\to SiH_{2}+SiH_{4}$$

(6b) $$2SiH_{3}\to Si_{2}H_{6}$$

(6c) $$SiH_{2}+SiH_{4}\to Si_{2}H_{6}$$

Then, the ArF excimer laser beam of 193 nm wavelength can be absorbed by disilane molecules which are produced by the plasma taking advantage of lower bond energy of disilane (~6.2 eV) [29]. The extinction of the laser with plasma in Figure 2c can be explained as the effect of generated disilane, and thus disilane molecules are decomposed by both of plasma and laser, also regenerating H and SiH_3_ radicals [29,34]. In the LAPECVD configuration, laser and plasma compete with each other, serving as sources to decompose disilane molecules as described by Equation (7) [34] in conjunction with a series of subsequent reactions. It is interesting to note the reduced laser extinction at the plasma power of 1000 W vs. at 600 W, indicating that the contribution of laser is lower when higher plasma power is applied, or the larger fraction of silane is decomposed by plasma of higher power. The dependence of laser extinction on silane pressure can be understood in the context of Equation (4), yet based on the partial pressure of disilane produced by plasma. However, it is important to note that in general the laser extinction in silane is much lower than that in ammonia even in plasma state, implying that the average concentration of disilane is much lower although the absorption cross section of disilane (~2 × 10^−15^ cm^2^) is much larger than that of ammonia (~1 × 10^−17^ cm^2^) for the illumination of 193 nm wavelength. This is because disilane molecules are products of finite life time generated in dynamic plasma states vs. ammonia molecules are reactants originally provided.

(7a) $$Si_{2}H_{6}+\left[h\nu \;or\;plasma\right]\to SiH_{4}+SiH+H$$

(7b) $$Si_{2}H_{6}+\left[h\nu \;or\;plasma\right]\to 2SiH_{2}+2H$$

(7c) $$Si_{2}H_{6}+\left[h\nu \;or\;plasma\right]\to 2SiH_{3}$$

(7d) $$Si_{2}H_{6}+\left[h\nu \;or\;plasma\right]\to Si\left(H_{2}\right)Si+4H$$

(7e) $$Si_{2}H_{6}+\left[h\nu \;or\;plasma\right]\to H_{2}SiSi+4H$$

**Figure 3:**
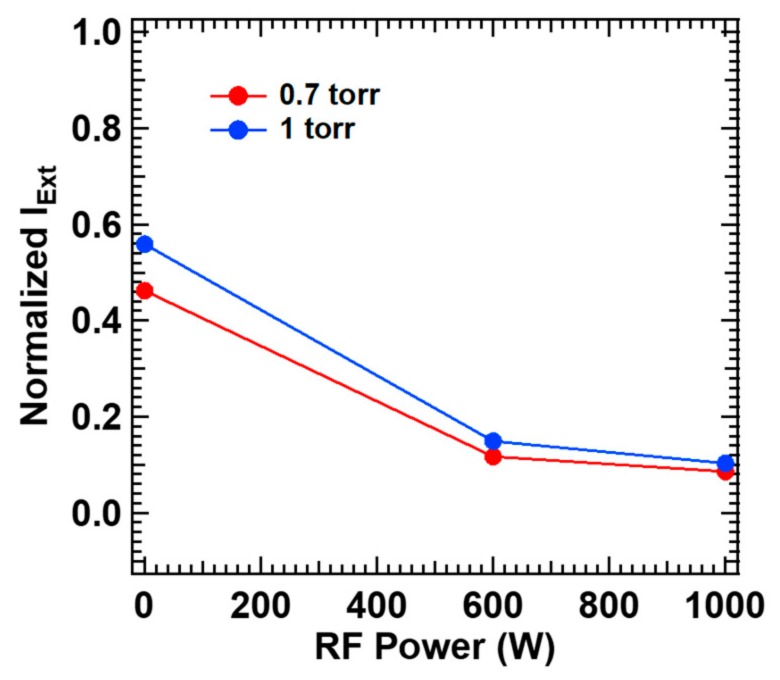
Normalized extinct laser power by propagation in the CVD chamber filled with N_2_, NH_3_ and SiH_4_ according to simultaneously driven RF power.

Finally, Figure 3 shows measured laser extinction with all the gases of NH_3_, SiH_4_ and N_2_ filled in, which corresponds to the reactant gas conditions to deposit SiN_x_ thin films by PECVD and LAPECVD in this study. Each of N_2_, NH_3_ and SiH_4_ was supplied through the gas shower at the flow rate of 150 sccm (i.e., 150 sccm for N_2_, 150 sccm for NH_3_ and 150 sccm for SiH_4_) with a separate N_2_ gas supplied to the laser window at 1000 sccm. Interestingly, the extinction profile for the gas mixture is similar to that for ammonia alone (Figure 2b), implying that the contribution of ammonia is still dominant even for the gas mixture. This is partly supported by the earlier investigations in Figure 2b,c although the contribution of silane cannot be fully neglected; definitely much higher laser extinction by ammonia at lower plasma power, and still ~2–3 times higher laser extinction by ammonia at higher plasma power. Initiated by the decomposition process explained so far, the radicals usually take part in subsequent aminosilane formation, surface condensation and termination to form silicon nitride thin film [28]. The decomposed NH_2_ and silicon hydrides produce aminosilane (SiH_x_(NH_2_)_y_) (x + y ≤ 4) based on the chemical reaction to substitute Si–H bonding of silicon hydride with Si-NH_2_ bonding and sequentially achieve higher number of y in aminosilane as described in Equation (8) [28,29]. The surface condensation occurs near the sample surface, i.e., condensation zone [29], where the amino radicals (NH_2_) are desorbed from aminosilane leading to outgassing in the form of ammonia (NH_3_) by hitching neighboring H atom while leaving Si, N and dangling bonds. The dangling bonds are developed into Si–N bonding that ultimately forms the silicon nitride network. The remaining radicals are terminated by combining with each other to become gaseous states. The surface condensation and termination steps are described in Equations (9) [29] and 10 [28], respectively. The extra laser absorption by the aminosilane cannot be excluded considering the low bond energy levels involved, possibly reducing defects or dangling bonds in the deposited silicon nitride film. Although a negligible effect on the laser extinction is anticipated due to the short life time, further investigation is needed.

(8a) $$SiH_{3}+NH_{2}\to SiH_{2}NH_{2}+H$$

(8b) $$SiH_{2}NH_{2}+H\to SiH_{3}NH_{2}$$

(8c) $$SiH_{2}NH_{2}+NH_{2}\to SiH{\left(NH_{2}\right)}_{2}+H$$

(8d) $$SiH_{3}NH_{2}+NH_{2}\to SiH_{2}{\left(NH_{2}\right)}_{2}+H$$

(8e) $$SiH{\left(NH_{2}\right)}_{2}+H\to SiH_{2}{\left(NH_{2}\right)}_{2}$$

(8f) $$SiH{\left(NH_{2}\right)}_{2}+NH_{2}\to Si{\left(NH_{2}\right)}_{3}+H$$

(8g) $$SiH_{2}{\left(NH_{2}\right)}_{2}+NH_{2}\to SiH{\left(NH_{2}\right)}_{3}+H$$

(8h) $$Si{\left(NH_{2}\right)}_{3}+NH_{2}\to Si{\left(NH_{2}\right)}_{4}$$

(8i) $$SiH{\left(NH_{2}\right)}_{3}+NH_{2}\to Si{\left(NH_{2}\right)}_{4}+H$$

(9) $$3Si{\left(NH_{2}\right)}_{4}+∆H\to Si_{3}N_{4}+8NH_{3}$$

(10a) $$2H\to H_{2}$$

(10b) $$H+NH_{2}\to +NH_{3}$$

**Figure 4:**
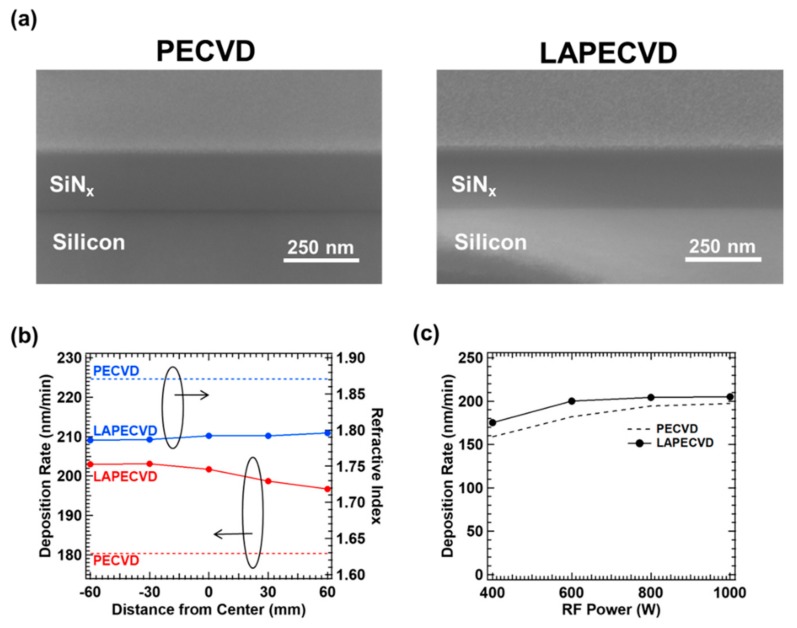
Cross sectional SEM images of silicon nitride thin films fabricated by (**a**) PECVD and LAPECVD, (**b**) deposition rate and refractive index of the silicon nitride thin films according to the distance from window and (**c**) thickness dependency of PECVD and LAPECVD according to power of RF power.

As next step, silicon nitride films deposited on silicon wafers by the aforementioned LAPECVD parameters were characterized. In order to evaluate the effect of laser illumination in LAPECVD, silicon nitride thin films deposited by similar PECVD conditions without laser illumination were also prepared as control samples. Each of N_2_, NH_3_ and SiH_4_ was supplied through the gas shower at the flow rate of 150 sccm with a separate N_2_ gas supplied to the laser window at 1000 sccm. The deposited film thickness for the deposition time of 1 min can be directly measured from the cross-sectional SEM image that also corresponds to the deposition rate per minute. The example cross sectional SEM images of silicon nitride thin films deposited by PECVD and LAPECVD by an identical plasma power of 600 W and the total reactant gas pressure of 0.7 torr for 1 minute, are shown in Figure 4a. The deposition thickness of 180 and 202 nm (or deposition rate in nm/min) was measured at the center location of the reaction chamber for PECVD and LAPECVD, respectively. Figure 4b displays the representative location dependence of the deposition rate and refractive index for the identical example deposition cases; PECVD and LAPECVD at plasma power of 600 W and the total pressure of 0.7 torr for 1 min. The relative position on the sample is specified in terms of the distance from the chamber center, and negative sign denotes the direction towards laser incidence window. By LAPECVD, an improved deposition rate was achieved vs. PECVD as an evidence of the laser’s contribution to the thin film deposition in the form of photolysis by 193 nm wavelength. While the deposition rate by PECVD was constant regardless of the relative position on the sample, that by LAPECVD decreases as laser beam propagates through reaction zone, which is consistent with the exponential decay trend in local laser intensity described by Equation (4), and the laser extinction measured in Figure 3. Further study is under way to achieve uniform deposition rate across larger sample area by tuning laser and PECVD parameters. On the contrary, the measured refractive index rather shows a slightly increasing trend along the laser beam propagation direction. The lower refractive index at the laser upstream in LAPECVD is an indicative of N-rich silicon nitride formation, thus supporting that the 193 nm laser illumination mainly decomposes NH_3_ in the excited state by plasma. Such N-rich trend has been also confirmed by the X-ray photoelectron spectroscopy (XPS). In this context, it is also reasonable that refractive index by LAPECVD is lower than that by PECVD; ~1.79 by LAPECVD vs. ~1.87 by PECVD. However, the variation in refractive index across the sample is relatively small, i.e., less than 0.01 over 120 mm sample dimension, which is a favorable aspect in a practical sense of encapsulating optoelectronic devices. Figure 4c shows the variation of deposition rate at the chamber center for a wider range of plasma power levels from 400 to 1000 W by PECVD and LAPECVD. As increasing the plasma power, the deposition rate was improved by both LAPECVD (from 175 to 203 nm/min) and PECVD (from 159 to 197 nm/min), and the deposition rate by LAPECVD was always higher than that by PECVD within tested plasma power range. However, an interesting trend is that the deposition rate is saturated at ~200 nm/min by LAPECVD, possibly indicating that majority of reactants, in particular ammonia, were decomposed by the plasma power of 600 W or larger at given gas pressure. Accordingly, the contribution of laser assisted decomposition gradually diminished as the plasma power increases beyond 600 W. Furthermore, based on these measurements for deposition rate, precursor yield for Si was calculated. The precursor yield of Si was 0.0541 for PECVD and 0.0568 for LAPECVD. The results showed that introduction of the laser during SiN_x_ thin films deposition improves the precursor yield.

**Figure 5:**
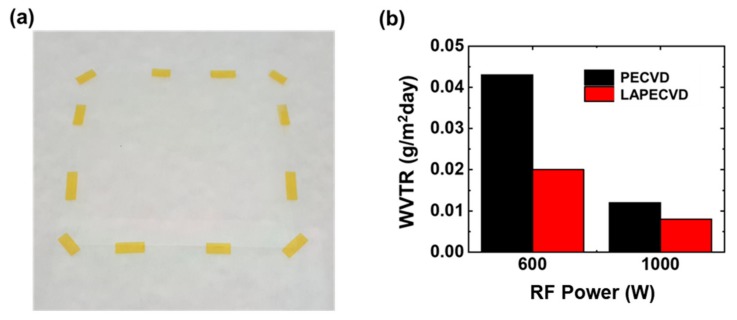
(**a**) Silicon nitride thin films fabricated onto 80 mm by 80 mm sized PEN substrate and (**b**) its water vapor transmission rate according to deposition methods of PECVD and LAPECVD.

Figure 5a is a photograph showing the silicon nitride thin films deposited on a PEN substrate of 80 mm × 80 mm size relying on LAPECVD (plasma power of 600 W) to evaluate the encapsulation performance by measuring the WVTR. A control sample was also prepared by PECVD (not shown here) using identical deposition parameters excluding laser illumination. Since the operating temperature limitation of the PEN film is higher than process temperature, both of the samples did not exhibit any thermal damage such as film shrinkage. The deposited thin films allowed excellent optical transparency with similar thickness and refractive index values vs. films on silicon wafers. As shown in Figure 5b, the measured WVTR after LAPECVD at the plasma power of 600 W was 0.043 g/m^2^ day and 0.020 g/m^2^ day for the sample deposited by PECVD and LAPECVD, respectively. The LAPECVD at the plasma power of 1000 W also improved the encapsulation performance although the improvement in 1000 W case was smaller than that in 600 W case, as expected. Enhanced encapsulation performance by LAPECVD is attributed to higher degree of decomposition of reactant species by the laser illumination in the vicinity of sample surface, continuously breaking bonds into the species of smaller molecular weight and thus forming films of reduced defects or dangling bonds. On the basis of the findings described above, a true merit anticipated for the LAPECVD configuration is in that excellent encapsulation performance is achievable at reduced plasma power and thus suppressing unwanted damage of underlying organic components by ion bombardment and/or ultraviolet emission of random orientation yet with equivalent or improved deposition rate vs. PECVD. Detailed studies in this regard are on-going.

## 4. Summary and Further Study

We deposited SiN_x_ thin films by LAPECVD based on use of an excimer laser of 193 nm wavelength for the encapsulation of PEN substrates at low substrate temperatures. The mechanisms of reactant gas decomposition were explored by measuring the laser extinction in individual and mixed gas conditions. In plasma states, the photolysis of ammonia is dominant, decomposing into NH_2_ and H radicals, while that of silane is relatively small as enabled by disilane that corresponds to the intermediate products generated by plasma excitation. In LAPECVD, the laser competes with or complements decomposition of reactant gases by plasma, and the laser generally plays a more important role at low plasma power. For a given plasma power and reactant pressure, laser coupling improved the deposition rate of SiNx thin films resulting in lower refractive indexes as an indication of N-rich composition due to predominant decomposition of ammonia by an excimer laser beam. The LAPECVD by laser of 193 nm wavelength also significantly improved the encapsulation performance as evaluated by the WVTR test, possibly due to a higher degree of decomposition of reactant species and thus the formation of films with reduced defects or dangling bonds upon coupling a laser beam. Further study is underway to improve the spatial uniformity in the deposition rate and to produce thin films of higher refractive index with minimal damage to the underlying organic components.

## Figures and Tables

**Table 1:** Experimental parameters of LAPECVD fabrication condition for measurement of *I_Ext_* and film thickness.

Experiment	Gas Flow				Process Condition			Laser Condition		
	SiH_4_ (sccm)	NH_3_ (sccm)	N_2_ (sccm)	N_2_ from Window (sccm)	W.P (mTorr)	Temperature (°C)	Time (s)	Beam Size (mm)	Pulse Energy (mJ)	Repetition Rate (Hz)
Experiment 1	0	0	750	1000	730	150	60	80 × 5	150	100
Experiment 2					1000					
Experiment 3	0	150	0	1000	730	150	60	80 × 5	150	100
Experiment 4					1000					
Experiment 5	150	0	0	1000	730	150	60	80 × 5	150	100
Experiment 6					1000					
Experiment 7	150	150	750	1000	730	150	60	80 × 5	150	100
Experiment 8					1000					
